# Surgical Treatment of Cerebellar Metastases: Survival Benefits, Complications and Timing Issues

**DOI:** 10.3390/cancers13215263

**Published:** 2021-10-20

**Authors:** Tunc Faik Ersoy, Neda Mokhtari, Daniel Brainman, Björn Berger, Attila Salay, Philipp Schütt, Florian Weissinger, Alexander Grote, Matthias Simon

**Affiliations:** 1 Department of Neurosurgery, Evangelisches Klinikum Bethel, Universitätsklinikum OWL, Burgsteig 13, 33617 Bielefeld, Germany; Tunc-Faik.Ersoy@evkb.de (T.F.E.); Neda.Mokhtari@evkb.de (N.M.); Daniel.Brainman@evkb.de (D.B.); Alexander.Grote@evkb.de (A.G.); 2 Department of Neuroradiology, Evangelisches Klinikum Bethel, Universitätsklinikum OWL, Burgsteig 13, 33617 Bielefeld, Germany; Bjoern.Berger@evkb.de; 3 Department of Radiotherapy, Brüderkrankenhaus St. Josef Paderborn, Husener Str. 46, 33098 Paderborn, Germany; a.salay@bk-paderborn.de; 4 Oncological Practice Gütersloh, Brunnenstraße 14, 33332 Gütersloh, Germany; schuett@onkologie-guetersloh.de; 5 Department of Hematology, Oncology and Palliative Care, Evangelisches Klinikum Bethel, Universitätsklinikum OWL, Schildescher Str. 99, 33611 Bielefeld, Germany; Florian.Weissinger@evkb.de

**Keywords:** cerebellar metastases, neurosurgery, complications, prognostic factors, survival

## Abstract

**Simple Summary:**

Cerebellar metastases are often considered to have a poor prognosis. This retrospective study investigated the clinical course and functional outcome of 73 patients who underwent surgical treatment for cerebellar metastases. Median overall survival was 9.2 months which compares favorably with the more recent literature. Prognosis varied strikingly between individuals. This suggests a policy of individualized decision-making which includes offering surgery also in selected cases with adverse prognostic parameters. The presence of extracerebral metastases did not significantly influence survival which may justify expedited surgery in selected cases prior to the oncological work-up. Systemic therapy was associated with substantially better survival indicating that recent advances in medical oncology might amplify any survival benefit derived from surgery. Surgery was found to carry significant morbidity and even mortality. Major complications often precluded adjuvant treatment and correlated with markedly reduced survival. Complication avoidance is therefore of utmost importance.

**Abstract:**

We retrospectively studied 73 consecutive patients who underwent surgery 2015–2020 for removal of cerebellar metastases (CM). Median overall survival (medOS) varied widely between patients and compared favorably with the more recent literature (9.2, 25–75% IQR: 3.2–21.7 months vs. 5–8 months). Prognostic factors included clinical (but not radiological) hydrocephalus (medOS 11.3 vs. 5.2 months, *p* = 0.0374). Of note, a third of the patients with a KPI <70% or multiple metastases survived >12 months. Chemotherapy played a prominent prognostic role (medOS 15.5 vs. 2.3, *p* < 0.0001) possibly reflecting advances in treating systemic vis-à-vis controlled CNS disease. Major neurological (≥30 days), surgical and medical complications (CTCAE III–V) were observed in 8.2%, 13.7%, and 9.6%, respectively. The occurrence of a major complication markedly reduced survival (10.7 vs. 2.5 months, *p* = 0.020). The presence of extracerebral metastases did not significantly influence OS. Postponing staging was not associated with more complications or shorter survival. Together these data argue for individualized decision making which includes offering surgery in selected cases with a presumably adverse prognosis and also occasional urgent operations in cases without a preoperative oncological work-up. Complication avoidance is of utmost importance.

## 1. Introduction

Surgical management of brain metastases plays an increasingly prominent role in neurosurgical practice. Brain metastases complicate the clinical course of >20% of cancers, and advances in medical oncology have resulted in a growing number of patients who are considered for neurosurgical care [1,2]. This includes cases with multiple metastases [3,4]. In some patients, the neurosurgeon aims at resecting all metastatic CNS disease while in others surgical treatment of selected tumors is performed as part of a multidisciplinary concept [1,2]. Resective treatment for brain metastases can be successfully combined e.g., with radiosurgery in order to provide aggressive local therapy for all metastatic deposits within the CNS [5,6]. Obtaining a tissue diagnosis and more recently tissues for molecular studies is also becoming more and more important [1,2,7,8].

Preserving neurological function and providing symptomatic relief is another indication for surgery of brain metastases. In particular, metastatic tumors of the posterior fossa come with a significant risk for neurological deterioration. If left untreated they will cause hydrocephalus, brainstem compression, and ultimately death. Many patients already present with signs and symptoms of hydrocephalus [9,10,11,12]. Most posterior fossa metastases are located within the cerebellar hemispheres and vermis. Cerebellar metastases account for approximately 20% of surgical cases with brain metastases [13].

Many neurosurgeons and neurooncologists feel that the neurological risks posed by the natural course of cerebellar metastases makes surgical treatment of these lesions somewhat urgent. Urgent or even emergency resections of cerebellar metastases will also treat any accompanying hydrocephalus, while temporary CSF diversion carries a very significant risk of meningitis, and permanent CSF shunts in patients with malignant brain tumors are associated with frequent complications [14,15]. On the other hand, an expedited surgical approach may preclude a complete oncological work-up and surgery is undertaken without full knowledge of the patient’s overall prognosis and chemo- and other systemic therapy options.

Surgery for cerebellar metastases may be very successful in terms of treating hydrocephalus and brainstem compression, but surgery for posterior fossa tumors also comes with a very significant morbidity and mortality. The literature contains surprisingly few pertinent datasets [16,17,18]. Complications may preclude further oncological therapy. This is a major and very significant argument against at least overly aggressive surgical management paradigms. In addition, survival after surgery even for single brain metastases may be poor with recent reports detailing a median survival of consistently below 12 months [19,20,21].

We felt that these issues together with the recent advances in medical oncology required a review of our current practice and possibly adjustments thereof. To this end we analyzed our institutional experience with surgery for cerebellar metastases in order to better define contemporary surgical indications with a view on an appropriate balance between surgical morbidity and mortality on the one hand, and patient survival on the other. We also attempted to address the question of timing of surgery. How important is the preoperative oncological work-up vis-a-vis the need for timely operations to prevent and treat hydrocephalus and brainstem compression?

## 2. Patients and Methods

### 2.1. Patients

We retrospectively identified all consecutive adult (>18 years) cases undergoing surgery for the removal of brain metastases from January 2015 to May 2020 in our department by searching the departmental electronic database. Patients operated for recurrent disease or receiving a biopsy (open or stereotactic) only were excluded. We reviewed the location of the respective growths which left *n* = 73 patients who had resective first surgery for cerebellar metastatic disease to be included in our study. Approval by the responsible institutional review board for human research and ethics committee was asked for and granted (Ethikkommission der Ärztekammer Westfalen-Lippe und der Westfälischen Wilhelms-Universität Münster, Münster Germany, Az 2021-073-f-S).

### 2.2. Surgical Indications

Throughout the study period we routinely offered surgery in cases with single metastatic tumors, but also in selected cases with multiple metastases whenever surgical removal of all tumors seemed safely possible through 1–2 craniotomies or as part of a multidisciplinary concept combining surgery with radiosurgery. Occasional patients had decompression of the posterior fossa only, i.e., removal of one or more large metastatic deposits in the cerebellum intentionally leaving macroscopic residual supra- or infratentorial disease behind for whole brain radio- and/or chemotherapy after carefully weighing the benefits and possible risks of surgery. Further indications included the need to obtain tumor tissue and a tissue diagnosis. Cases with growth into the brainstem had biopsies [22] and were not included in this study. Timing of surgery sometimes prioritized treatment and/or prevention of brainstem compression and hydrocephalus, i.e., staging studies and the oncological work-up were postponed until after the operation if expedited surgical treatment was deemed clinically beneficial.

### 2.3. Clinical and Radiological Data, Follow-Up

A chart review was performed to obtain all pertinent clinical data and follow-up information. We recorded age at surgery, gender, histopathological diagnosis, single vs. multiple tumors, additional infra- vs. supratentorial disease, overall number of tumors, meta- vs. synchronous presentation, primary tumor site and histology, and presence of extracranial metastases. GPA (graded prognostic assessment) scores were calculated for each patient [23]. We also noted the specifics of the metastasis surgery and hydrocephalus treatment. Additional parameters recorded include any preoperative oncological treatments, and the details of postoperative radio- and chemo- or other systemic therapy.

We studied overall survival as the primary oncological endpoint. Functional outcomes were assessed using the pre- and postsurgical (discharge) Karnofsky Performance Index (KPI). We also documented the details of all neurological, surgical, and medical complications occurring within 30 days of the index surgery. Complications were graded using the CTCAE classification (Common Terminology Criteria for Adverse Events v5.0; https://ctep.cancer.gov, accessed on 1 August 2021), and we distinguished between temporary neurological complications (<30 days) and neurological complications persisting beyond 30 days.

We also reviewed all pertinent radiological reports and imaging data. Preoperative MRI studies were available for all cases. We documented the location and number of all metastases, and the presence of hydrocephalus. Tumor load (index tumor/tumors, all cerebellar tumors, all metastatic tumors) was assessed by computer-assisted volumetric analyses using a well-established computer software (iplanNet, Brainlab AG, Munich, Germany). Postoperative neuroimaging (MRI: 36 [49.3%], CCT: 37 [50.7%]) was performed in all patients and within 24 h. in 71 (97.3%) cases. The respective radiological reports and scans were reviewed, and residual tumor and any complications were documented.

### 2.4. Statistical Analysis

We utilized commercially available software for statistical analysis (jamovi, Version 2.0, The jamovi project and IBM SPSS Statistics for Windows, Version 25.0, IBM Corp, Armonk, USA. Tests applied for univariate analysis were as follows: Fisher exact test, chi-square test, linear-by-linear association (Mantel–Haenszel test) and Student *t*-test. Two-sided tests were performed for all analysis. The significance limit was set at *p* < 0.05. Overall survival was studied using Kaplan–Meier estimates, median overall survival (OS) with 95% confidence intervals (95% CI), and the log rank test. For multivariate analyses, we used Cox regression modelling (inclusion procedure).

## 3. Results

### 3.1. Patient Cohort

Our series comprised 73 patients (60.3% females). Median age was 60.0 (25–75% IQR 53.5–70.0, range 30.0–82.0) years. The most frequent primary tumors were lung (50.7%) and breast cancer (26.0%). Thirty-four patients (46.6%) had multiple (2–3 tumors: *n* = 18, ≥4 tumors: *n* = 16) metastases. This included 26 cases with cerebellar and supratentorial disease (35.6%). Nineteen patients (26.0%) presented with synchronous and 54 (74.0%) with metachronous CNS metastases. In addition, 52 (71.2%; 1 missing) cases had extracranial metastases at the time of their index surgery. Volumetric analyses revealed a mean preoperative tumor volume of the index tumor of 15.9 ± 10.6 (median: 14.2, 25–75% IQR: 5.9–22.5) cm^3^. Mean cerebellar and overall CNS metastatic disease load was 16.3 ± 11.0 (median: 14.8, 25–75% IQR: 5.9–22.5) cm^3^ and 17.9 ± 13.2 (median: 15.3, 25–75% IQR: 6.5–27.8) cm^3^, respectively. Further characteristics of the study cohort are detailed in Table 1 and Table 2. Sixty cases were followed until death (82.2%), and median follow-up was 14.1 months in the remaining 13 patients still alive at the last follow-up.

### 3.2. Hydrocephalus Management and Surgical Treatment; Postoperative Radio- and Chemotherapy

Preoperative neuroimaging revealed obstructive hydrocephalus in 29 patients (39.7%). Sixteen (21.9%) cases also presented with clinical signs and symptoms of hydrocephalus. Three cases required external ventricular drains before and nine after their index surgery. Two of the 12 cases with ventricular drains vs. 0/61 without were treated for culture-positive bacterial meningitis (*p* = 0.03). One case had VP shunt placement surgery before the tumor resection. In two patients a permanent VP shunt was placed 44 and 72 days following the respective tumor surgery. Two of the three cases requiring CSF diversion had treatment for shunt infections during follow-up.

Seven cases (9.6%) presented with progressive disease following previous cranial radiotherapy and/or radiosurgery. Patients were operated for their cerebellar tumors in the prone (7 [9.6%]), lateral decubitus (5 [6.8%]) or sitting position (61 [83.6%]) depending on the tumor location and/or per surgeon’s preference. All cases were discussed in the interdisciplinary neuro-oncology tumor board and in additional disease-specific tumor boards as deemed necessary by the treating medical oncologist or radiotherapist. Fifty-three cases (72.6%) had postoperative radiotherapy, and 44 (61.1%, 1 missing) had postoperative chemo- or other systemic therapy. Multiple cerebellar metastases were addressed surgically in 11 cases, and four of these patients had additional craniotomies for supratentorial metastatic disease.

### 3.3. Complications and Functional Outcomes

The median preoperative KPI was 80% (IQR: 60–90), and the median postoperative KPI was also 80% (IQR: 60–90). The median postoperative KPI change was 0% (IQR: 0–10). Eighteen (22.5%) cases had a lower discharge than preoperative KPI. Conversely, the KPI improved in 30 (37.5%) cases following surgery. The 30 days mortality was 6.8% (5/73). This includes one patient dying from a cardiac complication, and two patients succumbing to complications of the primary disease unrelated to their index surgery.

New or aggravated major (CTCAE graded III–V) neurological deficits ≥30 days were observed in six (8.2%) cases (Table 3). Seven transient (<30 days) major deficits were seen in an additional five cases (6.8%) including early postoperative seizures [24] in two and somnolence resulting from pneumocephalus and/or hydrocephalus in four patients. Overall, ten patients (13.7%) incurred a CTCAE grades III–V surgical complication. This includes postoperative placement of ventricular drains for hydrocephalus in nine cases (12.3%). Four patients (5.5%) underwent revision surgery for a postoperative hemorrhage. There were two patients (2.7%) with culture-positive meningitis, and one case requiring surgery for a wound infection. Ten CTCAE grades III–V medical complications occurred in seven cases (9.6%). A detailed account can be found in Table 3.

The risk for incurring a major complication neurological deficit correlated with the preoperative KPI (KPI < 70%, 70–80%, 90–100%: 6/23 [26.1%], 2/25 [8.0%], 1/25 [4.0%]; *p* = 0.022), therefore also with the GPA score, and with tumor location (cerebellar hemispheres only vs. vermis involved: 3/62 [4.8%] vs. 3/11 [27.3%]; *p* = 0.04), but not with tumor volumetric findings, age, tumor multiplicity, presence of extracranial metastases or any other of the disease or patient characteristics assessed (Table 1). There were also statistically significant associations between the occurrence of a major medical complication and the preoperative KPI (KPI < 70%, 70–80%, 90–100%: 6/23 [26.1%], 1/25 [4.0%], 1/25 [4.0%]; *p* = 0.026) and tumor location (cerebellar hemispheres only vs. vermis involved: 3/62 [4.8%] vs. 4/11 [36.4%]; *p* = 0.008, Table 1). Tumor location also predicted surgical complications (cerebellar hemispheres only vs. vermis involved: 5/62 [8.1%] vs. 5/11 [45.5%]; *p* = 0.005, Table 1).

Complications often resulted in withholding radiotherapy, e.g., only 1/6 (16.7%) cases with a CTCAE grades III–V neurodeficit underwent radiotherapy vs. 52/67 (77.6%) cases without a grades III–V neurological complication (*p* = 0.005). Similarly, 1/6 (16.7%) vs. 43/66 (62.2%) patients with vs. without CTCAE neurodeficits had postoperative chemo- or other systemic therapy (*p* = 0.03, Table 1). Surgical complications had a lesser (negative) impact on the rates of postoperative radio- and/or systemic therapy than neurodeficits and medical complications (Table 1).

### 3.4. Patient Survival

**Figure 1:**
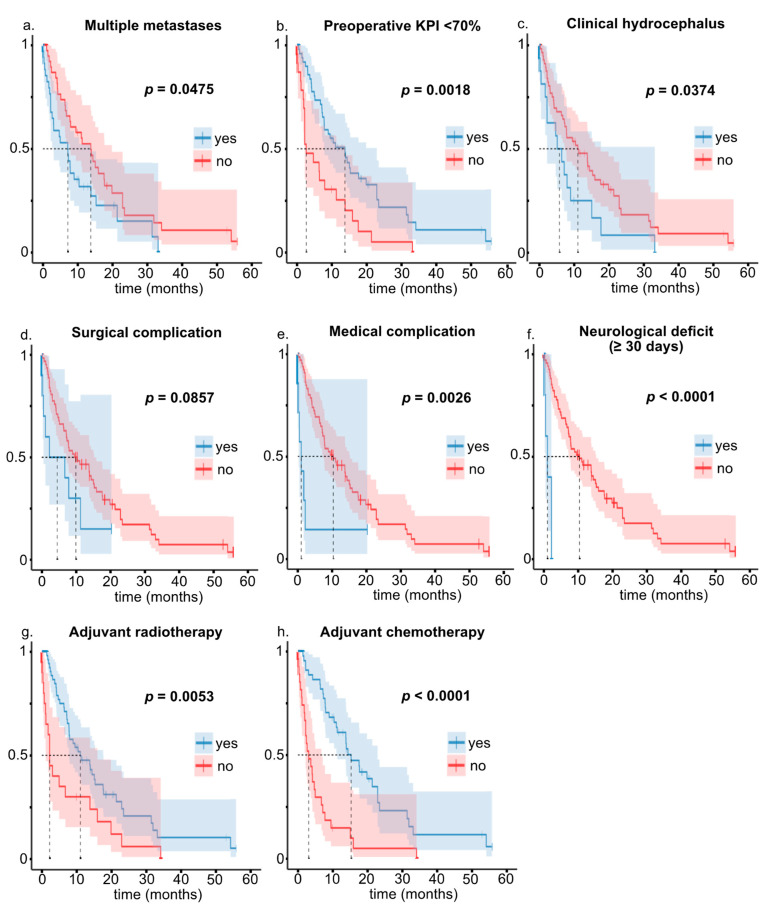
Kaplan–Meier estimates of overall survival stratified by prognostic parameters: (**a**) multiple metastases, (**b**) KPI < 70%, (**c**) clinical hydrocephalus, (**d**–**f**) major (CTCAE grades III–V) complications, (**g**) adjuvant radiotherapy, (**h**) adjuvant chemotherapy.

Median overall survival (OS) for the entire cohort was 9.2 months but varied strikingly between patients (25–75% IQR 3.2–21.7 months) and with various patient and disease characteristics (Table 2 and Figure 1). Median OS was 14.0 (25–75% IQR 5.2–23.4) months in cases with single and 7.3 (25–75% IQR 2.2–15.5) months in patients with multiple metastases (*p* = 0.0475). While median overall survival in 16 cases with ≥4 tumors was only 2.3 (25–75% IQR: 1.7–31.9) months, 5/16 (31.3%) survived their diagnosis by more than 12 months. Survival varied considerably with the patients’ KPI. Notably, this effect was most pronounced when using a KPI < 70% (median OS 2.7, 25–75% IQR 1.9–14.0 months) vs. KPI 70–100% (median OS 14.0, 25–75% IQR 5.2–23.9 months, *p* = 0.0018) cut-off. Still, 5/17 (29.4%) cases with a preoperative KPI < 70% survived their index surgery by >12 months. Clinical signs and symptoms of hydrocephalus proved to be a significant predictor of survival. Interestingly, age was not prognostic.

Pre- and postoperative tumor load (i.e., tumor volumetry findings) and additional supratentorial disease did not impact significantly on survival. Meta- vs. synchronous presentation and the presence of extracranial disease did not significantly correlate with the patients’ prognosis. Overall, parameters related to the extent and activity of the (primary) disease had surprisingly little influence on survival in this series (Table 2).

Complications heavily affected the patients’ prognosis (Table 2 and Figure 1). Median survival after incurring a major (CTCAE grades III–V) neurological, surgical, and/or medical complication was only ≤2.5 months, i.e., major complications following surgery for posterior fossa metastasis will usually preclude the patient from realizing any potential survival benefit resulting from the operation.

Lung cancer was the most common primary tumor and lung cancer patients were therefore also analyzed separately (Supplementary Table S1). We obtained some evidence that prognostic factors might vary with the primary cancer. Somewhat in contrast to the overall series, female sex correlated with better survival and volumetric findings were of borderline significance i.e., a larger tumor load predicted an adverse prognosis.

**Figure 2:**
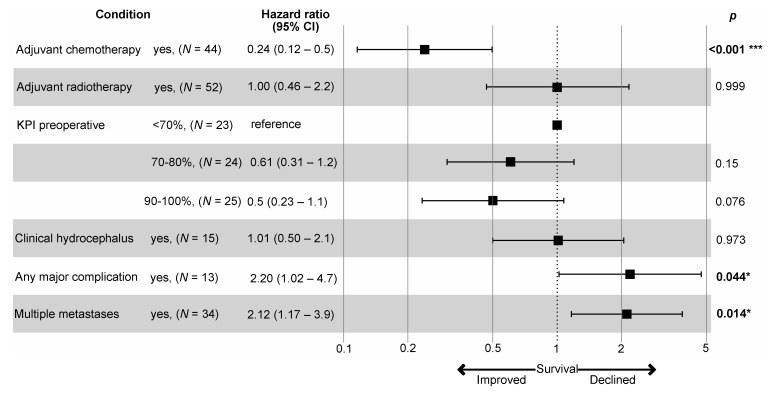
A multivariate Cox regression analyses of the prognostic factors for overall survival. Model metrics: R-squared = 0.397 (max. possible = 0.996), Likelihood ratio test = 36.374 (*p* < 0.001). *—significant, ***—highly significant.

A multivariate Cox regression analysis of the overall series with single vs. multiple metastases, preoperative KPI (<70%, 70–80%, 90–100%), clinical hydrocephalus, postoperative radiotherapy, postoperative chemo-/systemic therapy, and any CTCAE grades III–V complication as covariates revealed multiple metastases, no chemotherapy and incurring a major (CTCAE grades III–V) complication as independent negative prognostic factors for patient survival (Figure 2).

### 3.5. Pre- vs. Postoperative Staging

Nineteen cases (26.0%) underwent surgery prior to completion of staging and before a formal medical oncology consultation could be obtained. Median survival in this subset (8.1, 25–75% IQR 2.7–21.7 months) did not differ significantly from the remainder of our cohort (9.2, 25–75% IQR 3.7–20.2 months; *p* = NS). Rates of extracranial metastatic spread were similar (preoperative vs. postoperative staging: 13/18 [72.2%] vs. 39/54 [72.2%], *p* = NS). Complication rates (neurological CTCAE grades III–V deficits: 5/54 [9.3%] vs. 1/19 [5.3%], *p* = NS; CTCAE grads III–V surgical complications: 9/54 [16.7%] vs. 1/19 [5.3%], *p* = NS; CTCAE grades III–V medical complications: 6/54 [8.2%] vs. 1/19 [5.3%], *p* = NS) and the postoperative KPI (KPI < 70%, 70–80%, 90–100%: 18/54 [33.3%], 15/54 [27.8%], 21/54 [38.9%] vs. 1/19 [5.3%], 9/19 [47.4%], 9/19 [47.4%], *p* = NS) also did not vary significantly with completion of staging before vs. after the surgery.

## 4. Discussion

Surgical management of metastases accounts for an important and increasing part of the neurosurgical workload. Treatment of patients with cerebellar metastases may deserve to be studied separately [9,10,11,12,25]. The distribution of primary tumor histologies varies between posterior fossa and supratentorial metastases, i.e., the cerebellum is a predilection site for brain metastases from some (e.g., colorectal) cancers [1,26]. Here we report relatively good survival outcomes in a current cohort treated vis-à-vis recent advances in medical oncology. In addition, we provide a detailed account of complications using a well-established terminology and early postoperative functional outcomes.

Some groups reported a posterior fossa location as a negative prognostic factor in cases with brain metastasis [10,25,26]. Our data do not necessarily confirm worse survival in cases with cerebellar metastases when compared to brain metastases elsewhere. Median overall OS was 9.2 months in the present series, and 14.0 and 7.3 months in cases with single vs. multiple metastases. For comparison, in the large cohort published by Proescholdt et al. median overall survival was only 7.12 months [21]. In the cohort published by Pojskic et al. median overall survival was 8 and 6 months in cases with single and multiple brain metastases, respectively [27]. A recent publication details a median overall survival of 12 months in a retrospective cohort of 197 cases with single brain metastases not selected for location [20]. Loh et al. [28] describe a median overall survival of only 6.7, 6.8, and 10.5 months in patients with a GPA score of 0–1, 1.5–2.5 and 3–4 (cf. 2.3, 9.2, 15.3 months, present series). Our survival data also compare favorably with recently published cohorts detailing specifically the results of surgical management of cerebellar metastases. Sunderland et al. reported a median OS of only 6 vs. 5 months in patients with single vs. multiple including cerebellar metastases [10]. Median overall survival in the cohort published by Calluaud and co-workers was 7.9 months [11]. We would like to conclude that the survival outlook for patients with cerebellar metastases undergoing surgical treatment may not necessarily be as grim as often believed.

Importantly, survival after surgery for cerebellar metastasis appears to vary considerably with certain patient and tumor characteristics, but also between individual cases. This latter finding constitutes a significant challenge for surgical decision making, e.g., median survival in certain subgroups with adverse characteristics such as a KPI < 70% or more than three metastases was dismal (i.e., <3 months), however, OS was >12 months in approximately 30% of cases in both subgroups. We could not confirm the observation by others that vermal vs. hemispheric cerebellar tumor location impacts on survival [11]. The role of the degree of resection in brain metastasis surgery is controversial [19,20]. The high rate of complete resections in the present series may primarily reflect that 49.3% had postoperative CT rather than MR studies [11,19,20]. Synchronous vs. metachronous presentation was not prognostic in our series [10,11]. We obtained some albeit very tentative evidence that prognostic factors might vary with the histology of the primary tumor.

Postoperative systemic and radiotherapy heavily impacts on patient survival after surgery for brain metastases [1,10,11,20,21,25]. In our cohort, chemo- (or other systemic) therapy proved an important, very prominent, and in the multivariate analysis independent prognostic factor. Similar results have also been reported by others for all [20,21] and specifically for cerebellar metastasis [10]. Since the role of medical therapies in the treatment of CNS disease is still limited [2], it seems likely that the association between longer survival and systemic treatments points to the importance of controlling extracranial in addition to CNS disease. We report patients undergoing surgery from 2015 to 2020, while the cohort reported by Calluaud [11] consists of cases treated from 2007 through 2017, and the cases published by Sunderland were operated even earlier, i.e., between 2007 and 2012 [10]. Better survival in our cohort may therefore reflect to some extent advances in medical oncology in recent years. Postoperative complications also heavily impacted on patient survival in the present series. Complication and clinical outcome reporting in neurosurgery is an important topic and several classification schemes have been proposed [17,18,29,30,31]. However, no consensus has emerged so far. Somewhat similarly to Theodosopoulos et al. [30] we used the CTCAE classification to provide a detailed account of postoperative complications and their respective severity encountered in our patients. The CTCAE scheme is commonly used in oncological trials, and complications grades III–V are regarded as major and truly relevant. We found an overall 17.8% incidence of major (i.e., CTCAE grades III–V) complications in our patients. Major neurological deficits persisting ≥30 days were seen in 8.2%, and major surgical and medical complications in 13.7% and 9.6%, respectively. Based on pre- and postoperative KPI assessments more patients improved (37.5%) than deteriorated (22.5%) following surgery. Hadanny et al. report a 26.2% overall local (i.e., surgical) complication rate and slightly lower rates following osteoplastic craniotomies for posterior fossa metastases when compared to craniectomies [12]. However, this figure includes minor (CTCAE grade II) events. Mortality rates after surgery for cerebellar metastasis appear to be substantial (Sunderland et al.: 7.6% [10], Hadanny et al.: 2.3% [12], present series: 6.8%). Resective surgery does not always prevent patients from requiring permanent CSF diversion (Calluaud et al.: 0% [11], Chaichana et al.: 2% [25], present series: 4.1%, Sunderland et al.: 7.6% [10]).

There are also some reports describing in more detail outcomes and complication rates following surgery for brain metastases and brain tumors in general, e.g., Theodosopoulos and co-workers report a 10.3% major complication rate (using the CTCAE terminology) after surgery for intraaxial brain tumors [30]. Patel et al. describe a 3.3% mortality rate, 14.9% complications (major: 8.1%) and 4.9% major neurological deficits using a classification scheme, that is roughly comparable to the CTCAE terminology with a few (but especially for posterior fossa surgeries relevant) exceptions [18,29], e.g., EVD placement for hydrocephalus is not considered a major complication while it corresponds to a CTCAE grade III or even IV adverse event. Of note, in their initial 1998 publication the latter authors’ group describe more regional (i.e., surgical) and systemic, but not neurological complications after posterior fossa surgeries [18]. Taken together, these figures suggest higher overall but not necessarily neurological complication rates in patients with cerebellar vs. metastasis in other locations. Predicting complications in our cohort proved difficult. Similar to other investigators we found an association between a low KPI and the incurrence of neurological and medical complications. Somewhat surprisingly, increasing age was not significantly correlated with higher complicate rates [17,18,29]. Surgery for lesions involving the cerebellar vermis carried a higher risk for neurological, surgical, and medical complications.

Our data seem to provide some support for an expedited approach to surgery for cerebellar metastasis if felt necessary. Arguments in favor include the negative prognostic impact of clinical (but not radiological) hydrocephalus and of a low preoperative KPI, i.e., “early” surgery before functional deterioration may produce better oncological results. Managing (symptomatic) hydrocephalus in patients with posterior fossa metastases can be challenging [9]. Temporary as well as permanent CSF diversion for hydrocephalus was associated with significant complications in the present series. All three severe local infections (two cases with culture positive meningitis and one wound infection requiring surgical revision) were seen in patients with ventricular drains. Two of the three cases in this series requiring VP shunts developed shunt infections.

In addition, the rates of extracranial metastasis and—most importantly—survival as well as functional outcomes and complication rates did not differ significantly between cases with preoperative vs. postoperative staging. We do of course readily agree that our data clearly illustrate the major impact of postoperative treatment, i.e., postponing the oncological work-up implies surgical decision making vis-à-vis a far less than optimal assessment of the patient’s prognosis. Nevertheless, our data seem to show that urgent treatment of cerebellar metastasis—if deemed clinically necessary—will usually result in acceptable survival, complication rates, and functional outcomes.

The present analysis has substantial limitations. To name three significant ones, the cohort size does not allow for the investigation of more subtle effects and, e.g., cancer specific subgroups, and all data were obtained retrospectively, which is of course a particularly relevant concern when reporting and analyzing surgical indications, complications, and functional outcomes. Furthermore, the analysis of a surgical series is always associated with a considerable treatment selection bias. Operative treatment is usually offered not to all cases but primarily to patients believed to have a reasonable survival prognosis and who can tolerate the intervention.

## 5. Conclusions

First, our analysis suggests that survival after surgery for cerebellar metastasis varies considerably between individual patients and may generally be better than often thought. This may reflect that efficacious systemic therapeutic options are often increasingly available, which will amplify any survival benefit derived from surgery. Second, our data confirm that surgery for cerebellar metastasis comes with substantial risks and a significant mortality. Complication avoidance is of utmost importance when treating patients with CM. However, which patient will incur relevant complications is difficult to predict. Third, occasionally postponing the oncological work-up until after the surgery results in very acceptable outcomes.

Together, these findings seem to suggest a policy of individualized decision making which includes offering surgery in selected cases with a presumably adverse prognosis and occasional expedited or even urgent operations as the currently most appropriate neurosurgical treatment paradigm for cerebellar metastases.

## Figures and Tables

**Table 1:** Patient and metastases characteristics and neurological, surgical, and medical CTCAE grade III–V complications.

			Neurological Deficit ≥ 30 Days		Surgical Complication		Medical Complication	
		*n*	Yes	No	Yes	No	Yes	No
**Age**	≥60 yrs. (median)	37 (49.3%)	4 (10.9%)	33 (89.1%)	6 (16.3%)	31 (83.7%)	4 (10.9%)	33 (89.1%)
	<60 yrs.	36 (50.7%)	2 (5.6%)	34 (94.4%)	4 (11.2%)	32 (88.8%)	3 (8.4%)	33 (91.6%)
				*p* = 0.413		*p* = 0.525		*p* = 0.719
**Sex**	Female	44 (60.3%)	7 (15.9%)	37 (84.1%)	8 (18.2%)	36 (81.8%)	7 (15.9%)	37 (84.1%)
	Male	29 (39.7%)	2 (6.9%)	27 (93.1%)	5 (17.2%)	24 (82.8%)	1 (3.4%)	28 (96.6%)
				*p* = 0.303		*p* = 1.000		*p* = 0.135
**Preoperative KPI**	90–100%	25 (34.2%)	1 (4.0%)	24 (96.0%)	4 (16.0%)	21 (84%)	1 (4.0%)	24 (96.0%)
	70–80%	25 (34.2%)	2 (8.0%)	23 (92.0%)	2 (8.0%)	23 (92.0%)	1 (4.0%)	24 (96.0%)
	<70%	23 (31.5%)	6 (26.1%)	17 (73.9%)	7 (30.4%)	16 (69.6%)	6 (26.1%)	17 (73.9%)
				*p* = 0.022		*p* = 0.208		*p* = 0.026
**Clinical hydrocephalus**	Yes	16 (21.9%)	4 (25.0%)	12 (75.0%)	4 (25.0%)	12 (75.0%)	3 (18.8%)	13 (81.3%)
	No	57 (78.1%)	5 (8.8%)	52 (91.2%)	9 (15.8%)	48 (84.2%)	5 (8.8%)	52 (91.2%)
				*p* = 0.099		*p* = 0.463		*p* = 0.361
**Radiological hydrocephalus**	Yes	29 (39.7%)	3 (10.3%)	26 (89.7%)	7 (24.2%)	22 (75.8%)	4 (13.8%)	25 (86.2%)
	No	44 (60.3%)	3 (6.8%)	41 (93.2%)	3 (6.8%)	41 (93.2%)	3 (6.8%)	41 (93.2%)
				*p* = 0.591		*p* = 0.035		*p* = 0.322
**Cerebellar tumor location**	Hemispheres only	62 (84.9%)	3 (4.8%)	59 (95.2%)	5 (8.1%)	57 (91.9%)	3 (4.8%)	59 (95.2%)
	Vermis involved	11 (15.5%)	3 (27.3%)	8 (72.7%)	5 (45.5%)	6 (54.5%)	4 (36.4%)	7 (63.6%)
				*p* = 0.04		*p* = 0.005		*p* = 0.008
**Extent of CNS disease**	Single CM	39 (53.4%)	4 (10.3%)	35 (89.7%)	6 (15.4%)	33 (84.6%)	2 (5.1%)	37 (94.9%)
	Multiple metastases	34 (46.6%)	5 (14.7%)	29 (85.3%)	7 (20.6%)	27 (79.4%)	6 (17.6%)	28 (82.4%)
				*p* = 0.725		*p* = 0.760		*p* = 0.135
	Supratentorial disease: yes	26 (35.6%)	3 (11.5%)	23 (88.5%)	3 (11.5%)	23 (88.5%)	3 (11.5%)	23 (88.5%)
	~: no	47 (64.4%)	3 (6.4%)	44 (93.6%)	7 (14.9%)	40 (85.1%)	4 (8.5%)	43 (91.5%)
				*p* = 0.659		*p* = 1.000		*p* = 0.694
**Degree of resection (index tumor)**	Gross total	68 (93.2%)	5 (7.4%)	63 (92.6%)	9 (13.2%)	59 (86.8%)	6 (8.8%)	62 (91.2%)
	Subtotal	5 (6.8%)	1 (20.0%)	4 (80.0%)	1 (20.0%)	4 (80.0%)	1 (20.0%)	4 (80.0%)
				*p* = 0.357		*p* = 0.532		*p* = 0.405
**Postoperative tumor**	Yes	24 (32.9%)	4 (16.7%)	20 (83.3%)	4 (16.7%)	20 (83.3%)	4 (16.7%)	20 (83.3%)
	No	49 (67.1%)	2 (4.1%)	47 (95.9%)	6 (12.2%)	43 (87.8%)	3 (6.1%)	46 (93.9%)
				*p* = 0.086		*p* = 0.720		*p* = 0.208
**Volumetry ^1^**	Volume index tumor(s) ≥14.2 cm^3^ (median)	35 (50.0%)	3 (8.6%)	32 (91.4%)	6 (17.1%)	29 (82.9%)	4 (11.4%)	31 (88.6%)
	<14.2 cm^3^	35 (50.0%)	3 (8.6%)	32 (91.4%)	4 (11.4%)	31 (88.6%)	3 (8.6%)	32 (91.4%)
				*p* = 1.000		*p* = 0.734		*p* = 1.000
	Cerebellar tumor load ≥14.8 cm^3^ (median)	35 (50.0%)	3 (8.6%)	32 (91.4%)	6 (17.1%)	29 (82.9%)	4 (11.4%)	31 (88.6%)
	<14.8 cm^3^	35 (50.0%)	3 (8.6%)	32 (91.4%)	4 (11.4%)	31 (88.6%)	3 (8.6%)	32 (91.4%)
				*p* = 1.000		*p* = 0.734		*p* = 1.000
	Overall tumor load ≥15.3 cm^3^ (median)	35 (50.0%)	3 (8.6%)	32 (91.4%)	6 (17.1%)	29 (82.9%)	4 (11.4%)	31 (88.6%)
	<15.3 cm^3^	35 (50.0%)	3 (8.6%)	32 (91.4%)	4 (11.4%)	31 (88.6%)	3 (8.6%)	32 (91.4%)
				*p* = 1.000		*p* = 0.734		*p* = 1.000
**Presentation**	Synchronous	19 (26.0%)	1 (5.3%)	18 (94.7%)	1 (5.3%)	18 (94.7%)	1 (5.3%)	18 (94.7%)
	Metachronous	54 (74.0%)	5 (9.0%)	49 (91.0%)	10 (19.0%)	44 (81.0%)	7 (13.0%)	47 (87.0%)
				*p* = 0.585		*p* = 0.164		*p* = 0.355
**Primary tumor site**	Lung	37 (50.7%)	3 (8.1%)	34 (91.9%)	4 (10.8%)	33 (89.2%)	3 (8.1%)	34 (91.9%)
	Breast	19 (26.0%)	5 (26.3%)	14 (73.7%)	7 (36.8%)	12 (63.2%)	5 (26.3%)	14 (73.7%)
	Gastrointestinal tract	9 (12.3%)	1 (5.9%)	16 (94.1%)	2 (11.8%)	15 (88.2%)	0	17 (100%)
	Renal	3 (4.1%)						
	Melanoma	2 (2.7%)						
	Other	3 (4.1%)						
				*p* = 0.840		*p* = 0.561		*p* = 0.712
**Extracranial metastases ^2^**	Yes	52 (72.2%)	8 (15.4%)	44 (84.6%)	11 (21.2%)	41 (78.8%)	7 (13.5%)	45 (86.5%)
	No	20 (27.8%)	1 (5.0%)	19 (95.0%)	2 (10.0%)	18 (90.0%)	1 (5.0%)	19 (95.0%)
				*p* = 0.429		*p* = 0.330		*p* = 0.429
**Radiotherapy**	Yes	53 (72.6%)	1 (1.9%)	52 (98.1%)	4 (7.5%)	49 (92.5%)	1 (1.9%)	52 (98.1%)
	No	20 (27.4%)	5 (25.0%)	15 (75.0%)	6 (30.0%)	14 (70.0%)	6 (30.0%)	14 (70.0%)
				*p* = 0.005		*p* = 0.021		*p* = 0.001
**Chemo-/systemic therapy ^2^**	Yes	44 (61.1%)	1 (2.3%)	43 (97.7%)	4 (9.1%)	40 (90.9%)	1 (2.3%)	43 (97.7%)
	No	28 (38.9%)	5 (17.9%)	23 (82.1%)	6 (21.4%)	22 (78.6%)	6 (21.4%)	22 (78.6%)
				*p* = 0.03		*p* = 0.172		*p* = 0.012
**GPA score ^2^**	0–1.0	20 (27.8%)	6 (30.0%)	14 (70.0%)	6 (30.0%)	14 (70.0%)	5 (25.0%)	15 (75.0%)
	1.5–2.5	39 (54.2%)	2 (5.1%)	37 (94.9%)	5 (12.8%)	34 (87.2%)	2 (5.1%)	37 (94.9%)
	3.0	8 (11.1%)	1 (7.7%)	12 (92.3%)	2 (15.4%)	11 (84.6%)	1 (7.7%)	12 (92.3%)
	3.5–4.0	5 (6.9%)						
				*p* = 0.029		*p* = 0.214		*p* = 0.073

Abbreviations: CTCAE—common terminology criteria for adverse events, yrs.—years, KPI—Karnofsky performance index, CNS—central nervous system, CM—cerebellar metastasis, GPA—graded prognostic assessment. ^1^: volumetric data from three patients could not be made available, ^2^: data from one case are missing.

**Table 2:** Patient and metastases characteristics as possible predictors of patient survival.

		*n*	OS (Months)	95%CI (Months)	*p* (Log Rank Test)
**Age**	≥60 yrs. (median)	37 (49.3%)	7.9	6.2–9.7	0.727
	<60 yrs.	36 (50.7%)	10.7	3.9–17.5	
**Sex**	Female	44 (60.3%)	11.5	3.4–19.7	0.063
	Male	29 (39.7%)	8.1	4.3–11.8	
**Preoperative KPI**	90–100%	25 (34.2%)	14.2	9.1–19.2	0.007
	70–80%	25 (34.2%)	10.7	2.4–19.0	
	<70%	23 (31.5%)	2.7	0.4–5.1	
**Clinical hydrocephalus**	Yes	16 (21.9%)	5.2	1.6–8.8	0.037
	No	57 (78.1%)	11.3	4.6–18.0	
**Radiological hydrocephalus**	Yes	29 (39.7%)	8.1	6.6–9.5	0.071
	No	44 (60.3%)	14.0	7.3–20.7	
**Cerebellar tumor location**	Hemispheres only	62 (84.9%)	9.2	5.6–12.8	0.988
	Vermis involved	11 (15.5%)	66.9	0.6–13.3	
**Extent of CNS disease**	Single CM	39 (53.4%)	14.0	9.4–18.6	0.047
	Multiple metastases	34 (46.6%)	7.3	3.5–11.1	
	Supratentorial disease: yes	26 (35.6%)	7.3	2.0–12.6	0.095
	~: no	47 (64.4%)	11.5	5.3–17.7	
**Degree of resection (index tumor/~s)**	Gross total	68 (93.2%)	8.1	4.5–11.8	0.314
	Subtotal	5 (6.8%)	14.0	0–37.7	
**Any postoperative CNS tumor**	Yes	24 (32.9%)	7.3	0.5–14.1	0.136
	No	49 (67.1%)	11.3	4.8–17.7	
**Volumetry ^1^**	Volume index tumor(s) ≥14.2 cm^3^ (median)	35 (50.0%)	11.3	2.8–19.8	0.983
	<14.2 cm^3^	35 (50.0%)	9.2	6.0–12.4	
	Cerebellar tumor load ≥14.8 cm^3^ (median)	35 (50.0%)	11.3	3.7–18.8	0.902
	<14.8 cm^3^	35 (50.0%)	7.9	4.5–11.3	
	Overall tumor load ≥15.3 cm^3^ (median)	35 (50.0%)	11.5	3.2–19.7	0.558
	<15.3 cm^3^	35 (50.0%)	7.9	6.5–9.3	
**Presentation**	Synchronous	19 (26.0%)	7.4	2.2–12.5	0.419
	Metachronous	54 (74.0%)	9.7	6.3–13.1	
**Primary tumor site**	Lung	37 (50.7%)	7.4	3.0–11.7	0.088
	Breast	19 (26.0%)	6.9	0–14.0	
	Gastrointestinal tract	9 (12.3%)	16.1	11.7–20.6	
	Renal	3 (4.1%)			
	Melanoma	2 (2.7%)			
	Other	3 (4.1%)			
**Extracranial metastases ^2^**	Yes	52 (72.2%)	7.9	2.6–13.2	0.321
	No	20 (27.8%)	10.7	2.0–19.3	
**Radiotherapy**	Yes	53 (72.6%)	11.3	5.9–16.6	0.005
	No	20 (27.4%)	2.3	2.2–2.4	
**Chemo-/systemic therapy ^2^**	Yes	44 (61.1%)	15.5	10.2–20.9	<0.0001
	No	28 (38.9%)	3.2	1.2–5.2	
**GPA score ^2^**	0–1.0	20 (27.8%)	2.3	0–5.4	0.059
	1.5–2.5	39 (54.2%)	9.2	5.2–13.1	
	3.0	8 (11.1%)	15.3	5.9–24.6	
	3.5–4.0	5 (6.9%)			
**Complications (CTCAE grades III–V)**	Surgical: yes	10 (13.7%)	2.5	0–11.3	0.085
	~: no	63 (86.3%)	10.7	3.2–18.1	
	Neurological (≥30 days): yes	6 (8.2%)	1.1	0–3.1	<0.0001
	~: no	67 (91.8%)	10.7	4.3–17.1	
	Medical: yes	7 (9.6%)	1.1	0–2.6	0.002
	~: no	66 (90.4%)	10.7	4.4–16.9	
	Any	13 (17.8%)	2.5	0–7.9	0.020
	None	60 (82.2%)	10.7	3.7–17.6	

Abbreviations: OS—median overall survival, CI—confidence interval, yrs.—years, KPI—Karnofsky performance index, CNS—central nervous system, CM—cerebellar metastasis, GPA—graded prognostic assessment, CTCAE—common terminology criteria for adverse events. ^1^: volumetric data from three patients could not be made available, ^2^: data from one case are missing.

**Table 3:** CTCAE grades III–V complications following surgery for cerebellar metastases.

	Complications	CTCAE			
		III	IV	V	III–V
**Neurological ^1^**	Confusion	2			2
	Depressed level of consciousness			2	2
	Dizziness	2			2
	Dysphagia	2			2
	**Patients**				6
**Surgical**	Intracranial hemorrhage	1	4		5
	Hydrocephalus	1	9		10
	Meningitis	1	1		2
	Wound infection	1			1
	Pneumocephalus		1		1
	**Patients**				10
**Medical**	Acute kidney injury	1			1
	Anemia	1			1
	Asystole			1	1
	Atelectasis		1		1
	Laryngeal edema		1		1
	Lung infection	1	2		3
	Pneumothorax		1		1
	**Patients**				7

Abbreviations: CTCAE—common terminology criteria for adverse events. ^1^: ≥30 days.

## Data Availability

The datasets generated during and/or analyzed during the current study are available from the corresponding author on reasonable request.

## Supplementary Materials

The following are available online at www.mdpi.com/article/10.3390/cancers13215263/s1, Table S1: Patient and metastases characteristics as possible predictors of lung cancer patient survival.
