# Supplementary material for: Surgical Treatment of Cerebellar Metastases: Survival Benefits, Complications and Timing Issues
Source: Cancers (Basel). 2021 Oct 20;13(21):5263. doi: 10.3390/cancers13215263 (PMC8582465; doi:10.3390/cancers13215263)
Supplement: Supplementary file 1 [file cancers-13-05263-s001.zip › cancers-1392591-supplementary.pdf]

Supplementary Table S1. Patient and metastases characteristics as possible predictors of lung cancer patient survival.

		n	OS (Months)	95%CI (Months)	*p* (log rank test)
**Age**	≥60 yrs. (median)	19 (51.4%)	4.3	1.4–7.2	0.800
	<60 yrs.	18 (48.6%)	7.4	5.8–9.0	
**Sex**	Female	16 (43.2%)	15.3	1.8–28.7	0.007
	Male	21 (56.8%)	4.3	1.2–7.4	
**Preoperative KPI**	90–100%	14 (37.8%) [3]	9.7	0–20.6	0.021
	70–80%	13 (35.1%) [3]	5.2	0.2–10.2	
	<70%	10 (27.0%) [3]	2.3	2.2–2.4	
**Clinical hydrocephalus**	Yes	7 (18.9%)	4.8	2.4–12.5	0.151
	No	30 (81.1%)	7.4	0–11.1	
**Radiological hydrocephalus**	Yes	12 (32.4%)	5.2	0.7–9.7	0.099
	No	25 (67.6%)	7.4	2.8–11.9	
**Cerebellar tumor location**	Hemispheres only	33 (89.2%)	7.4	3.7–11.1	0.094
	Vermis involved	4 (10.8%)	2.3	0.9–3.7	
**Extent of CNS disease**	Single CM	17 (45.9%)	9.7	0–21.5	0.092
	Multiple metastases	20 (54.1%)	1.8	0–6.8	
	Supratentorial disease: yes	16 (43.2%)	3.2	0–11.5	0.341
	~: no	21 (56.8%)	8.1	3.0–13.1	
**Degree of resection (index tumor/~s)**	Gross total	34 (91.9%)	5.2	0.7–9.7	0.991
	Subtotal	3 (8.1%)	14.0	0–31.4	
**Any postoperative CNS tumor**	Yes	12 (32.4%)	2.3	0–8.2	0.515
	No	25 (67.6%)	7.4	2.0–12.8	

**Volumetry [1]**	Volume index tumor(s) ≥14.2 cm³ (median)	17 (45.9%)	4.3	2.2–6.4	0.050
	<14.2 cm³	18 (54.1%)	9.2	3.2–6.4	
	Cerebellar tumor load ≥14.8 cm³ (median)	17 (45.9%)	4.3	2.2–6.4	0.050
	<14.8 cm³	18 (54.1%)	7.9	3.2–12.6	
	Overall tumor load ≥15.3 cm³	16 (36.7%)	4.3	2.2–6.4	0.050
	<15.3 cm³	19 (63.3%)	7.9	3.2–12.6	
**Presentation**	Synchronous	17 (45.9%)	7.4	2.5–12.3	0.478
	Metachronous	20 (54.1%)	4.8	0–11.6	
**Extracranial metastases [2]**	Yes	21 (59.5%)	3.2	0.8–5.6	0.244
	No	15 (40.5%)	10.6	3.8–17.5	
**Radiotherapy**	Yes	31 (83.8%)	8.1	5.5–10.6	<0.0001
	No	6 (16.2%)	2.3	0–2.6	
**Chemo-/ systemic therapy [2]**	Yes	23 (62.2%)	10.7	1.8–19.5	0.001
	No	13 (37.8%)	3.2	1.7–3.8	
**GPA score [2]**	0-1.0	9 (25.0%)	2.2	1.6–2.9	0.163
	1.5–2.5	19 (52.8%)	7.4	3.6–11.2	
	3.0	4 (11.1%)	14.2	12.4–15.9	
	3.5–4.0	4 (11.1%)			
**Complications (CTCAE grades III–V)**	Surgical: yes	3 (8.1%)	2.5	2.3–4.2	0.092
	~: no	34 (91.9%)	7.4	2.8–11.9	
	Neurological (≥30 days): yes	3 (8.1%)	2.2	0.5–4.0	<0.0001

	~: no	34 (91.9%)	7.4	3.3–11.5	
	Medical: yes	2 (5.4%)	1.1	NA	0.003
	~: no	35 (94.6%)	7.4	3.6–11.3	
	Any	4 (10.8%)	2.2	1.1–3.4	0.024
	None	33 (89.2%)	7.4	3.6–11.2	

Abbreviations: OS-median overall survival, CI—confidence interval, yrs.—years, KPI—Karnofsky performance index, CNS—central nervous system, CM—cerebellar metastasis, GPA—graded prognostic assessment, CTCAE—common terminology criteria for adverse events, NA—not applicable. [1]: volumetric data from two patients could not be made available, [2]: data from one case are missing, [3]: percentages do not add up to 100% because of rounding errors.
